# Effects and Mechanisms of Lutein on Aging and Age-Related Diseases

**DOI:** 10.3390/antiox13091114

**Published:** 2024-09-14

**Authors:** Jialu Ye, Jin Cheng, Ruogu Xiong, Haoqi Chen, Siyu Huang, Huabin Li, Jinzhu Pang, Xuguang Zhang, Huilian Zhu

**Affiliations:** 1 Department of Nutrition, School of Public Health, Sun Yat-sen University, Guangzhou 510080, China; yejlu@mail2.sysu.edu.cn (J.Y.); chengj225@mail2.sysu.edu.cn (J.C.); xiongrg@mail2.sysu.edu.cn (R.X.); chenhq55@mail2.sysu.edu.cn (H.C.); huangsy9@mail2.sysu.edu.cn (S.H.); lihuabin@mail.sysu.edu.cn (H.L.); 2 Mengniu Institute of Nutrition Science, Global R&D Innovation Center, Inner Mongolia Mengniu Dairy (Group) Co., Ltd., Hohhot City 011050, China; pangjinzhu@mengniu.cn (J.P.); fxgzhang@mengniu.cn (X.Z.); 3 Sun Yat-sen University-Mengniu Joint Research Center of Nutrition and Health for Middle-Aged and Elderly, School of Public Health, Sun Yat-sen University, Guangzhou 510080, China

**Keywords:** lutein, aging, age-related diseases, age-related macular degeneration, cataract, Alzheimer’s disease, Parkinson’s disease, osteoporosis

## Abstract

Aging and age-related diseases are serious public health issues that are receiving growing attention from researchers. Lutein has a critical function in the prevention and management of these issues. Possible mechanisms mainly include suppressing inflammation and oxidative stress, regulating cell activity, and modulating the levels of toxic substances. In this narrative review paper, we sum up the most current developments in the study of the effects of lutein on aging and five age-related diseases (age-related macular degeneration, cataracts, Alzheimer’s disease, Parkinson’s disease, and osteoporosis), and fundamental mechanisms are reviewed. The bioavailability of lutein and the strategies to improve its bioavailability are discussed. This piece of work can bring a clearer comprehension of the protective effects of lutein against aging and age-related diseases and can be also helpful for developing lutein as functional food and dietary supplements for these age-related diseases.

## 1. Introduction

Aging, a complicated procedure defined by the progressive accumulation of a wide variety of molecular and cellular damage, will lead to functional decline and a decrease in physical and mental capacity [1]. The aging of the population is a heavy burden for the world. According to the World Health Organization, the number of people over 60 will rise from one billion in 2020 to 1.4 billion by 2030 and 2.1 billion by 2050 [2]. The functional disability of aging is directly associated with the progression of multiple age-related diseases [3,4]. For example, age-related macular degeneration is a major reason for blindness, which has negative impacts on millions of individuals [5]. Therefore, slowing down the aging process and maintaining functional ability are important for prolonging the lifespan, improving life quality, and lowering the burden of the healthcare system.

Although there are several theories to explain the nature of the aging process, the free radical theory of aging is the most popular theory as a conceivable explanation of the aging process proposed by Denham Harman in 1956 [6]. It holds that the accumulation of oxidative stress, which is mainly produced through oxidative respiration by the mitochondrial electron transport chain, could cause pathophysiological alterations, functional decline, and accelerated aging and many age-related diseases. Additionally, cell senescence is related to the activation of many pathways. For example, p21 is regarded as one of the primary senescent cell regulators and indicators that is mainly transactivated via p53 [7]. The activation of the p53–p21 pathway could induce cell death and inhibit cell growth. Cells that express high levels of p21 (p21^high^ cells) show increased expression in the NF-κB signaling pathway, chemotaxis, and inflammatory response [8]. Thus, anti-aging drugs have drawn much attention, such as metformin, rapamycin, resveratrol, and senolytics [9]. Antioxidants have also been suggested as having a preventive effect against age-related diseases and aging. As a powerful antioxidant, lutein has beneficial effects in delaying aging and preventing age-related diseases [10]. Therefore, in this narrative review paper, the effects and mechanisms of lutein on aging and age-related diseases are summarized and discussed given the outcomes of epidemiological, experimental, and clinical studies. This review could be helpful for lutein to be developed into functional food and dietary supplements for the prevention and management of these age-related diseases.

## 2. Effects and Mechanisms of Lutein on Aging

**Figure 1:**
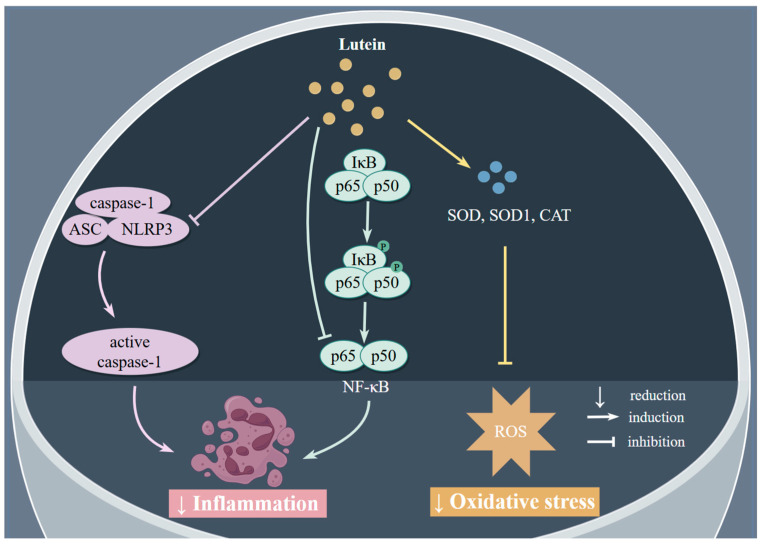
Effects and mechanisms of lutein on aging. Mechanisms involved are downregulation of NF-κB pathway and NLRP3 and upregulation of antioxidant enzymes. ASC, apoptosis-associated speck-like protein containing CARD; CAT, catalase; IκB, inhibitor of NF-κB; NF-κB, nuclear factor kappa-B; NLRP3, NOD-like receptor thermal protein domain-associated protein 3; ROS, reactive oxygen species; SOD, superoxide dismutase. Figure by Figdraw.com (accessed on 27 July 2024).

Aging is triggered by several hallmarks such as genomic instability, telomere attrition, epigenetic alterations, the loss of proteostasis, disabled macroautophagy, deregulated nutrient-sensing, mitochondrial dysfunction, cellular senescence, stem cell exhaustion, etc. [11]. Accumulating evidence from epidemiological, experimental, and clinical studies has suggested that lutein may play a vital role in slowing the aging process. The mechanisms of lutein in aging are discussed in detail below (Figure 1).

### 2.1. Epidemiological Studies

Lutein intake was found to be associated with some aging-related biomarkers. Telomere length is an aging biomarker, and its shortening is thought to be connected to aging and age-related diseases. A cross-sectional cohort study found that a higher lutein level in plasma was related to longer telomere length in elderly adults (β = 0.079, *p* = 0.03, adjusted for age and sex) [12]. Moreover, the *Klotho* gene is thought to exhibit anti-aging potential and suppress cell senescence, and soluble klotho (S-klotho) is a circulating protein inducing multiple protective effects and may be a potential cure for aging and age-related diseases. One cross-sectional study found that the total carotenoid intake was positively related to S-klotho levels in serum, and higher lutein intake was also related to higher S-klotho levels in serum [13]. Moreover, lutein was associated with a lower risk of age-related diseases. Meta-analysis revealed that dietary lutein consumption was inversely correlated with the risks of various age-related diseases such as macular degeneration, cataracts, coronary heart disease, stroke, and esophageal cancer [14]. The details of epidemiological studies are shown in Table 1.

To sum up, most of the epidemiological studies found that a higher intake or serum levels of lutein were related to lower risks of age-related diseases and associated with the upregulation of anti-aging biomarkers and the downregulation of aging biomarkers.

### 2.2. Experimental Studies

Various studies have pointed out that lutein has protective effects against aging, and its basic mechanisms include its antioxidant and anti-inflammatory effects. The details of experimental studies are shown in Table 2.

#### 2.2.1. Antioxidant Effects

Compared to the basal diet group, there were increases in the survival time and antioxidant enzyme activity, such as that of superoxide dismutase (SOD) and catalase (CAT), and a decrease in the peroxidation product malondialdehyde (MDA) level in *Drosophila melanogaster* with a diet containing lutein [44]. In addition, excessive radiation can cause an upregulated level of oxidative stress and then induce the aging process. An in vivo study found that lutein exhibited a protective effect against oxidative damage caused by electron beam radiation in Swiss albino mice by mitigating oxidative changes and maintaining the balance of the antioxidant system [45]. Furthermore, microcystin-LR can promote the generation of reactive oxygen species (ROS) and oxidative stress and trigger mutations, resulting in DNA damage and genetic instability, which is the hallmark of aging. Lutein treatment inhibited the production of ROS and the reduction in CAT expression, as well as the survival loss induced by microcystin-LR in *Caenorhabditis elegans* (*C. elegans*) [46]. Another study discovered that lutein treatment could increase resistance to heat and oxidative stress and extend the health span of *C. elegans* and *Drosophila* cells [47].

#### 2.2.2. Anti-Inflammatory Effects

Persistent inflammation was thought to be harmful and related to the process of aging and multiple age-related diseases [48]. In several experimental studies, lutein exhibited anti-aging capacities through its anti-inflammatory effect. An in vitro experiment showed that lutein could effectively alleviate the senescence of mesenchymal stem cells (MSCs) through suppressing the inflammation of senescent MSCs and showed powerful anti-aging potential in other tissues and organs probably by downregulating the levels of ROS and inflammation, and the underlying mechanisms were the downregulation of nuclear factor kappa-B (NF-κB) and NOD-like receptor thermal protein domain-associated protein 3 (NLRP3) and the upregulation of the *Clock* gene’s expression [49].

In brief, lutein showed anti-aging potential by increasing antioxidant capacity and decreasing the damage of oxidative stress and alleviating inflammation.

**Table 2:** Experimental studies of lutein on aging and age-related diseases.

Study Type	Model	Dose and Duration	Effects and Mechanisms	Ref.
Aging				
In vivo	*D. melanogaster*, wild-type, Oregon-R-C	0.03, and 0.1 mg lutein/mL	↑ mean lifespan ↓ MDA ↑ antioxidant enzyme activities ↑ expression of SOD1, SOD2, and CAT	[44]
In vivo	Swiss albino mice	5, 50, 100, and 250 mg/kg b.wt for 15 days	↑ survival time ↑ TAC in lung, brain, and liver ↑ CAT activity ↑ glutathione in brain and lung ↓ MDA protected liver and kidney function	[45]
In vivo	*C. elegans*	1, 10, 100, 250, and 500 µg/L for 30 min	↓ ROS ↓ CAT ↓ survival loss	[46]
In vivo	*C. elegans*	10 and 100 µM	↑ survival rate ↑ lifespan ↓ ROS ↑ CAT, neuroligin 1	[47]
In vitro and in vivo	Mesenchymal stem cells	10, 20, 30, 50, and 100 µM	↑ growth rate, cell proliferation, cell viability ↓ SA-β-gal-positive cells ↓ p21, p16, and p53 ↑ expression of *Clock* gene ↓ TNF-α, IL-1β, and IL-6 ↓ NF-κB and NLRP3 ↓ ROS, MDA	[49]
AMD				
In vitro	H_2_O_2_-induced ARPE-19 cells	5, 10, and 20 µM for 3 days	↓ ROS ↓ production of SA-β-gal ↓ G2 arrest ↑ HO-1 and NQO1 ↑ activation of Nrf2 ↓ p53–p21 pathway	[50]
In vitro	H_2_O_2_-induced ARPE-19 cells	0, 2.5, 5, 10, 20, and 40 µM for 24 h	↓ omega-6 PUFA oxidation ↓ pro-inflammatory HETE ↓ Isop ↓ transcriptional regulation of GPx and NFE2L2	[51]
In vivo	Male Wistar rats	39 nmol/d for 8 weeks	↓ VEGF ↑ SOD2 ↓ abnormalities in ganglion cell and diabetic retina ↓ mRNA expression of *Hif1α* and *Xbp1*	[52]
In vivo	Male SD rats	25, 50, and 100 mg/kg body weight for 30 days	Attenuated decrease in electroretinogram a-wave and b-wave amplitudes and thinning of photoreceptor cell layer caused by apoptosis ↓ light-induced oxidative stress ↓ inflammatory cytokine levels ↑ expression of BCO2	[53]
In vitro	Human primary corneal epithelial cells (HCE-F)	50, 100, and 250 µM	↓ ROS ↓ apoptotic cell death ↑ Nrf2, ratio of Nrf2/Keap1 ↓ Keap1	[54]
In vitro	NIH/3T3 Swiss albino mouse fibroblast cells	0, 0.01, 0.1, 1, and 10 µM lutein for 6 h	↓ ROS	[55]
In vivo	Abca4^(−/−)^/orAbca4^(−/−)^/Bco2^(−/−) ^ double-knockout C57BL/6 mice	1 g/kg of diet for 3 months	↓ A2E and iso-A2E ↑ visual performance	[56]
In vitro	Rat Muller cells	2.5, 5, 10, and 20 µM for 24 h	↑ cell viability ↓ cell apoptosis ↑ Bcl-2/Bax ratio ↓ caspase-3 ↓ LC3II ↓ autophagosome formation ↑ p-mTOR/mTOR	[57]
In vitro	ARPE cells	0.1, 0.5, 1.5, and 10 µM for 24 h	↓ expression of *TXNIP*, *CXCL8*, *BAX*, *CASP1* ↑ expression of *BCL2*	[58]
In vitro	ARPE-19 cells	1 µM	↓ ERS ↑ IRE1-XBP1 pathway ↑ ATF6 ↑ ATF4	[59]
Cataract				
In vitro	Human lens epithelial cells	5 µM for 48 h	↓ protein carbonyl ↓ MDA ↓ DNA damage ↑ GSH and GSH: GSSG ratio ↓ H_2_O_2_-induced cell death	[60]
In vivo and in vitro	Shumiya cataract rats and human lens epithelial cells	In vivo: 2 mg/kg body weight for 3 weeks In vitro: 5, 10 µM for 48 h	↓ mRNA levels of peroxiredoxin 6 and catalase in both models	[61]
In vitro	Human lens epithelial cells	2 mmol/L for 4 h	↓ JNK, p38 ↓ lipid peroxidation	[62]
In vivo	Type 1 diabetic rat	Short-term: 10 mg/kg body weight for 29 days Long-term: 0.4 mg/kg body weight for 69 days	↓ N-epsilon-(carboxymethyl)lysine ↓ N-epsilon-(carboxyethyl)lysine	[63]
AD				
In vivo	Male Wistar rats	5 mg/kg body weight daily for 1 month	↓ MDA ↓ total oxidative status ↑ TAC ↑ passive avoidance learning, spatial memory in Morris water maze and Barnes maze tests, and cognitive memory	[64]
In vivo	Zebrafish/female mice	Zebrafish: 0.93, 1.56 mg/L for 10 days Mice: 285, 668 mg/kg for 10 days	↑ escape spatial learning and memory ↓ brain AChE activity ↑ glutathione ↑ activity of SOD	[65]
In vivo	Wistar rats	100 mg/kg for 8 weeks	↓ MDA ↑ antioxidant enzyme activities ↑ Nrf2 and HO-1 ↓ NF-κB	[66]
In vitro	Cerebrovascular endothelial cells	0.8 µM for 12 h	↑ cell viability ↓ ROS and lipid peroxides ↓ NF-κB ↑ Nrf2, NQO1, and HO-1 ↓ apoptosis	[67]
In vitro	BV-2 cells	2.5, 5, 7.5, and 10 ng/µL for 24 h	↓ ROS ↓ IL-1β, TNF-α ↑ IL-4	[68]
In vitro	SH-SY5Y cells	2.5, 5, 7.5, and 10 ng/µL for 24 h	↓ ROS ↓ CAT activity ↓ TNF-α, IL-6, IL-8 ↓ HAMP ↓ Glu-induced accumulation of iron ↓ lipoxygenases	[69]
In vitro	Rat PC-12 cells	0.2, 2, 20, and 200 µM for 2 h	↑ cell viability ↓ ROS ↓ apoptosis ↑ Bcl-2 ↓ active caspase-3/7 level ↓ MAPK pathways (pERK1/2, p-p38, p-JNK)	[70]
In vivo	Male C57BL/6 mice	5, 10, and 20 mg/kg body weight/day for 7 days	↓ loss of nigral dopaminergic neurons ↑ striatal dopamine level ↓ MPTP-induced mitochondrial dysfunction ↓ oxidative stress and motor abnormalities ↓ MPTP-induced neuronal damage/apoptosis ↓ pro-apoptotic markers (Bax, caspases-3, 8,9) ↑ anti-apoptotic marker (Bcl-2)	[71]
In vitro	PC12 cells	5, 10, 20 µM for 2 h	↓ oxidative damage and apoptosis ↓ *caspase-3*, *caspase-9*, *Bax*c-caspase-3 ↑ Bcl-2/Bax ratio, *Bcl-2* ↑ *PI3K, Akt* PI3K inhibitor abolished protective effect of lutein	[72]
In vitro	PC12 cells	20 µM for 2 h	↓ H_2_O_2_-mediated growth inhibition and morphological changes ↓ mRNA expression of *AMAD10* and *Bax* ↓ phosphorylation of JNK1/2	[73]
In vitro	SH-SY5Y cells	0.1, 1, and 10 µM for 24 h	↑ glutathione ↓ ROS Protected against mitochondrial uncoupling	[74]
In vivo	*C. elegans*	1 µM for 6 days	↓ neurodevelopmental deficits Restored mitochondrial dysfunction-induced neuroligin expression	[75]
In vivo	Female Sprague-Dawley rats	50 or 100 mg/kg for 14 days	↑ body weight ↑ neurobehavioral alterations ↑ attenuated oxidative stress ↑ mitochondrial enzyme complex activities of rat brain Neuroprotective effect	[76]
In vitro	SH-SY5Y cells	5 µM for 72 h	↑ differentiation of SH-SY5Y cells ↑ pAkt ↑ microtubule-associated protein 2 ↑ ROS ↑ glucose consumption, rates of glycolysis ↑ respiratory activity of mitochondrial complexes ↑ acetyl-CoA, PDH expression, HK activity	[77]
In vitro		10, 20, and 50 µM for 24 h	↓ Aβ fibril formation	[78]
In vivo	Wistar rats	50 mg/kg for 14 days	Reversed memory deficit ↓ activity of AChE	[79]
PD				
In vivo	Rotenone-induced *Drosophila melanogaster*	6 µM for 7 days	↑ survival rate ↑ dopamine levels ↑ tyrosine hydroxylase ↑ activity of AchE ↑ SOD, CAT activity ↓ thiobarbituric acid reactive substances and glutathione S-transferase	[80]
In vivo	Male C57BL/6 mice	5, 10, and 20 mg/kg body weight/day for 7 days	↓ loss of nigral dopaminergic neurons ↑ striatal dopamine level ↓ MPTP-induced mitochondrial dysfunction ↓ oxidative stress and motor abnormalities ↓ MPTP-induced neuronal damage/apoptosis ↓ pro-apoptotic markers (Bax, caspases-3, 8,9) ↑ anti-apoptotic marker (Bcl-2)	[71]
Osteoporosis				
In vivo	Ovariectomized Wistar rats	50 mg/kg for 4 weeks	↓ serum lipid peroxide and glutathione ↓ femur tissue lipid peroxide and ROS ↑ CAT, SOD, GST, GPx ↓ IL-6, IL-8, TNF-α ↓ NF-κB, IL-6, NFATc1 ↑ Nrf2, NQO1, HO-1	[81]
In vitro	Primary rat chondrocytes	1 µM for 24 h	Protective effect against cytotoxicity ↓ oxidative stress ↑ SOD, CAT, GST, GPx ↑ Nrf2, HO-1, and NQO1	[82]
In vivo and in vitro	Newborn and 5- and 6-week-old *ddy* mice Primary osteoblastic cells, bone marrow cells	In vitro: 3 and 10 µM for 14 days In vivo: 66 mg/d for 4 weeks	↑ formation of mineralized bone nodules ↓ 1α, 25-dihydroxy vitamin D3-induced bone resorption ↓ 1α, 25-dihydroxy vitamin D3-induced osteoclast formation ↓ RANKL ↑ osteoclast formation ↓ femoral bone mass in cortical bone in vivo	[83]
In vitro	Primary osteoblastic cells	3, 10, and 30 µM for 24 h	↓ expression of RANKL in osteoblasts ↓ IL-1-induced osteoclast formation and bone resorption ↓ macrophage differentiation into osteoclasts ↓ mature osteoclast survival ↑ bone formation (↑ *BMP2* ↓ *Sclerostin*)	[84]
In vitro	Mononuclear cells of mouse bone marrow	10^−8^, 10^−7^, and 10^−6^ mol/L for 7 days	↓ number of osteoclast cells ↓ TRAP activity ↓ percentage of bone surface ↑ expression of RANK ↓ osteoclast differentiation in vitro	[85]
In vitro	Femoral diaphyseal and femoral metaphyseal tissues of male Wistar rats	10^−8^–10^−6^ M for 48 h	↓ metaphyseal alkaline phosphatase activity	[86]

Abbreviations: A2E, N-retinylidene-N-retinylethanolamine; Aβ, amyloid beta protein; AChE, acetylcholinesterase; Akt, serine/threonine protein kinase; ATF, activating transcription factor; Bax, BCL2-associated X; Bcl-2, B-cell lymphoma-2; BCO2, beta-carotene oxygenase 2; BMP2, bone morphogenetic protein 2; CAT, catalase; CLOCK, clock circadian regulator; CXCL8, C-X-C Motif Chemokine Ligand 8; ERS, endoplasmic reticulum stress; GPx, glutathione peroxidase; GSSG, glutathione, oxidized; GST, glutathione S-transferase; HAMP, hepcidin antimicrobial peptide; HETE, hydroxyeicosatetraenoic acid; HIF-1α, hypoxia-inducible factor 1; HK, hexokinase; HO-1, heme oxygenase 1; IL, interleukin; IRE1, inositol-requiring enzyme 1; IsoP, iso-prostane; Keap1, Kelch-like ECH-associated protein 1; LC, light chain; MAPKs, mitogen-activated protein kinases; MDA, malondialdehyde; MPTP, 1-methyl-4-phenyl-1,2,3,6-tetrahydropyridine; mTOR, mammalian target of rapamycin; NF-κB, nuclear factor kappa-B; NFATC1, nuclear factor of activated T cells 1; NFE2L2, nuclear factor, erythroid 2-like 2; NLRP3, NLR pyrin domain protein 3; NQO1, NAD(P)H quinone oxidoreductase 1; pAkt, phosphorylated Akt; pERK, Phospho-extracellular regulated protein kinase; p-JNK, Phospho-Jun N-terminal kinase; PDH, pyruvate dehydrogenase; PI3K, phosphoinositide 3-kinase; RANK, receptor activator of nuclear factor-κB; RANKL, receptor activator of nuclear factor-κB ligand; ROS, reactive oxygen species; SA-β-gal, senescence-associated beta-galactosidase; SOD, superoxide dismutase; TAC, total antioxidant capacity; TNF-α, tumor necrosis factor alpha; TRAP, tartrate-resistant acid phosphatase; TXNIP, Thioredoxin-Interacting Protein; VEGF, vascular endothelial growth factor; XBP1, X-box binding protein 1. ↑, upregulation; ↓, downregulation.

## 3. Effects and Mechanisms of Lutein on Age-Related Diseases

Many researchers have found that there are beneficial effects of lutein on age-related diseases. In this section, the effects and mechanisms of lutein on the five most common age-related diseases, including age-related macular degeneration, cataract, Alzheimer’s disease, Parkinson’s diseases, and osteoporosis, are summarized and discussed.

### 3.1. Lutein and Age-Related Macular Degeneration (AMD)

**Figure 2:**
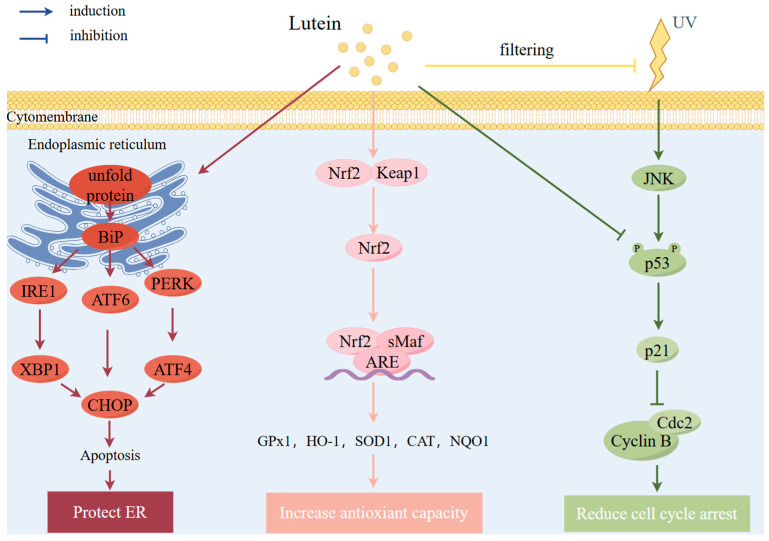
Effects and mechanisms of lutein on AMD. Mechanisms involved are activation of Nrf2 pathway, filtering UV, downregulation of p53–p21 pathway, and regulation of ERS. AMD, age-related macular degeneration; ARE, antioxidant response element; ATF4, activating transcription factor 4; ATF6, activating transcription factor 6; BiP, glucose-regulated protein (GRP78); CAT, catalase; Cdc, cell division cycle; CHOP, C/EBP homologous protein; ER, endoplasmic reticulum; GPx1, glutathione peroxidase 1; HO-1, heme oxygenase 1; IRE1, inositol-requiring transmembrane kinase endoribonuclease-1; JNK, c-Jun N-terminal kinase; Keap1, Kelch-like ECH-associated protein 1; NQO1, NAD(P)H quinone oxidoreductase 1; Nrf2, nuclear factor erythroid 2-related factor 2; PERK, protein kinase R-like ER kinase; sMaf, small Maf transcription factor; SOD1, superoxide dismutase 1; UV, ultraviolet; XBP1, X-box binding protein 1. Figure by Figdraw.com (accessed on 27 July 2024).

AMD is the degeneration of the central area of the retina. Due to continuous oxidative stress, byproducts of the visual cycle accumulate in retinal pigment epithelium (RPE) cells; induce cell injury, the dysregulation of RPE function, and abnormalities in extracellular matrix deposition; and eventually lead to complement activation and a series of inflammation, photoreceptor cell death, and the loss of vision [87]. Lutein plays a vital role in human vision across the whole lifespan. At the very earliest stages of retinal embryology, lutein and zeaxanthin start to accumulate in the vitreous humor [88]. Additionally, only two (lutein and zeaxanthin) of the many carotenoids in human serum and diet are found in the retina, and these carotenoids form a yellow pigmentation in the central retina yields which is known as “macular pigment (MP)” [89], and its optical density (MPOD) is the standard unit of measurement for the concentration of MP, and it is related to visual performancesuch as vision accuracy, photo stress recovery, contrast sensitivity, the remission of glare disability and glare disability, and dark-adapted visual sensitivity [90,91,92,93]. Moreover, there are quite a number of relevant studies suggesting that lutein could reduce the risk of the onset and progression of AMD through several mechanisms. The mechanisms of lutein in AMD are discussed in detail below (Figure 2 and Table 2).

#### 3.1.1. Epidemiological Studies

Many epidemiological studies have indicated that lutein intake is inversely associated with the risk of AMD. For example, a case–control study and meta-analysis revealed that higher concentrations of lutein and zeaxanthin in plasma were associated with a lower risk of AMD (OR = 0.21, 95% CI = 0.05, 0.84) [15]. Moreover, a cross-sectional study revealed that MPOD was significantly higher in early AMD patients than in advanced AMD patients [16]. Also, a cohort study discovered a correlation between higher plasma lutein and a 37% reduced risk of advanced AMD [17]. Another case–control study found that the consumption of lutein and lutein-rich foods was inversely associated with the risk of AMD [18]. A cohort study found that a higher lutein/zeaxanthin intake was correlated with a lower risk of AMD progression [19]. However, another case–control study showed that in the non-AMD group, the average lutein/zeaxanthin intake was similar to that in the AMD group, probably due to the insufficient lutein intake of both groups [20]. The details of epidemiological studies are shown in Table 1.

In brief, most epidemiological studies suggested that a higher lutein intake had protective effects against both AMD morbidity and its progression. Because inconsistent results were also reported, more epidemiological studies with a bigger sample size, stricter study design, and representative groups should be conducted in the future.

#### 3.1.2. Experimental Studies

Lutein may prevent the progression of AMD caused by peroxidation and photo-damage mainly through several mechanisms, such as its protective effects against light irradiation, antioxidant effects, reducing the toxic substance level, and downregulating endoplasmic reticulum (ER) stress, which will be discussed in detail below. The details of experimental studies are shown in Table 2.

##### Antioxidant Effects

Aging and age-related oxidative damage play a crucial role in the pathophysiology of AMD, in addition to other genetic and environmental variables [94]. A number of studies have found that lutein may contribute to AMD prevention through antioxidant action. Several studies revealed that lutein could downregulate the oxidative stress in arising human RPE (ARPE) cells. For example, one experimental study showed that lutein reduced the ROS and SA-β-gal levels and upregulated the expression of heme oxygenase 1 (HO-1) and NAD(P)H quinone dehydrogenase 1 and downregulated the p53–p21 pathway in H_2_O_2_-induced ARPE-19 cells [50]. Moreover, lutein and zeaxanthin treatment could regulate inflammatory lipid mediators caused by oxidative stress and reduce pro-inflammatory HETE in H_2_O_2_-induced ARPE-19 cells [51]. Additionally, vascular endothelial growth factor (VEGF) is significant in AMD progression, and one 8-week experiment found that lutein downregulated VEGF probably through decreasing the mRNA expression of *Hif1α* and *Xbp1* genes, upregulating SOD2 in the retina of diabetic rats; and ameliorating abnormalities in ganglion cells and the inner and outer nuclear layers [52].

##### Protective Effects against Light Irradiation

Retinal degeneration can be induced by exposing the eyes to intense sunlight or UV light that is focused on the lens and retina. Of all visible light, 440 nm blue light damages the retina with 100 times less energy than 590 nm orange light [95]. One retrospective study observed less progression of geographic atrophy in individuals with blue light-filtering intraocular lenses than in those without color filters [96]. Due to its structure, lutein can absorb blue light and therefore protect the eye structure from its radiation. For example, a study found that mice exposed to light in a yellow intraocular lens material box (a material that has the same filtration effect as lutein) showed a lower expression of pro-inflammatory cytokines, ROS levels, and macrophage recruitment in the RPE–choroid compared to mice exposed in clear material [97]. Moreover, lutein pretreatment prevented oxidative stress caused by light in the retinal tissues, downregulated the levels of inflammatory cytokines, and significantly attenuated the apoptotic-induced thinning of the photoreceptor cell layer and decrease in electroretinogram a- and b-wave amplitudes in rats [53]. A study showed that lutein protected human primary corneal epithelial cells from blue-violet light phototoxicity through scavenging ROS, inhibiting apoptotic cell death, and regulating the nuclear factor erythroid 2-related factor 2 (Nrf2) pathway [54]. Furthermore, lutein/zeaxanthin treatment protected photoreceptors from light damage both morphologically and functionally by ameliorating oxidative stress and ER stress in light-induced *Pde6^rd10^* mice [98]. Moreover, lutein reduced the levels of ROS produced in mammal cells with blue light irradiation treatment [55].

##### Other Mechanisms

One study found that a lutein and zeaxanthin supplement decreased the levels of N-retinylidene-N-retinylethanolamine (A2E) and iso-A2E, which is the content of lipofuscin in the RPE of *Abca4^(−/−)^*/*Bco2^(−/−)^* double-knockout mice [56]. Furthermore, lutein could improve cell survival by the regulation of apoptosis and autophagy pathways in cobalt(II) chloride-treated Muller cells [57]. Moreover, lutein treatment in ARPE-19 cells showed that lutein regulated the expression of pyroptosis-related genes such as *TXNIP*, *CXCL8*, *BAX*, and *CASP1* [58]. Furthermore, lutein could reduce hyperglycemia-mediated ER stress in ARPE-19 cells by triggering the inositol-requiring enzyme 1(IRE1) -XBP1, activating transcription factor 4 (ATF4), and ATF6 pathways and their downstream activators [59]. Another experimental study found that senolytic drug ABT-263, which shows inhibitive effects on Bcl-2 and Bcl-xL, selectively triggered apoptosis in senescent ARPE-19 cells [99].

In summary, experimental studies suggested that lutein could prevent the occurrence and progression of AMD through ameliorating oxidative stress, protecting the retina from phototoxicity especially caused by blue light irradiation, decreasing lipofuscin levels, and regulating cell death.

#### 3.1.3. Clinical Trials

Many clinical trials have suggested that lutein supplements can postpone AMD progression and improve visual performances in AMD patients. For example, a randomized, double-blinded, placebo-controlled trial found that lutein supplementation for 2 years raised MPOD, serum lutein levels, and visual sensitivity in individuals with early AMD [100]. Another randomized, double-masked, placebo-controlled trial revealed that lutein supplementation for 1 year could increase MPOD and contrast sensitivity in early AMD patients [101]. Moreover, a randomized, double-blind, placebo-controlled, two-center investigation found that lutein supplementation increased MPOD and showed mild improvement in visual acuity (VA) in early AMD patients with lutein supplementation for 1 year [102].

The Age-Related Eye Disease Study (AREDS) 2 is a study aimed at determining whether adding lutein/zeaxanthin and other components to the AREDS formulation reduces the hazard of developing advanced AMD and other age-related eye diseases. Although AREDS2 revealed that no significant decrease was observed in the risk of advanced AMD for lutein/zeaxanthin, subgroup analyses showed that for participants in the lowest quintile of dietary intake (mean = 696 µg/day), lutein/zeaxanthin exhibited protective effects against AMD progression possibly because the lutein/zeaxanthin consumption of the individuals was similar to that of other well-nourished groups [103]. Additionally, a post hoc analysis of two controlled clinical trial cohorts AREDS and AREDS2 found that dietary lutein consumption was inversely correlated with the risk of AMD progression [104]. However, one study found that there was no significant effect of 6-month lutein supplementation in AMD patients on the mean differential light threshold (MDLT) or VA, but a significant correlation was observed between the rise in MPOD and the rise in MDLT (r = 0.25, *p* = 0.027) or VA (r = 0.27, *p* = 0.013) after 6 months, suggesting that patients who had a significant rise in MPOD when taking lutein also saw an improvement in their visual function [105]. The results of several clinical trials are summarized in Table 3.

In short, most clinical studies suggested that lutein supplementation could improve visual performance or slow AMD progression. Because inconsistent results were also reported, more clinical studies with a bigger sample size, stricter study design, and representative groups should be conducted in the future.

### 3.2. Lutein and Age-Related Cataracts

Cataracts are characterized as crystalline lens opacities that can result in blindness, which has become a serious public health issue. It is commonly recognized that during the development of age-related cataracts, oxidative stress causes biochemical alterations in the components of the lens [113]. Lutein is a strong antioxidant with the function of filtering blue light, and many studies showed that lutein play a role in preventing and improving cataract [114].

#### 3.2.1. Epidemiological Studies

A cross-sectional study discovered that older individuals with higher lutein concentrations in plasma have a reduced risk of age-related cataracts (RR = 0.58, 95% CI = 0.35–0.98, *p* = 0.041) [21]. Additionally, a cohort study revealed that lutein/zeaxanthin consumption was inversely correlated with the prevalence of nuclear opacification [22]. A meta-analysis including one cohort study and seven cross-sectional studies revealed that the blood concentration of lutein/zeaxanthin was inversely associated with the risk of nuclear cataracts [23]. A dose–response meta-analysis containing six cohort studies found that there was a significant inverse correlation between lutein/zeaxanthin intake and the risk of age-related cataracts. Moreover, every 300 µg/d increase in dietary lutein and zeaxanthin intake was linked to a 3% decrease in the risk of nuclear cataracts, according to a dose–response analysis [24]. Another dose–response meta-analysis containing eight RCTs and twelve cohort studies also showed that dietary lutein/zeaxanthin consumption was inversely correlated with the risk of age-related cataracts (RR = 0.81, 95% CI = 0.75–0.89, *p* < 0.001), and a dose–response analysis found that every 10 mg/d increase in dietary lutein and zeaxanthin intake was linked to a 26% decrease in the risk of age-related cataracts [25]. The details of epidemiological studies are shown in Table 1.

In brief, most epidemiological studies supported the idea that a higher intake and blood concentration of lutein is associated with a lower risk of age-related cataracts.

#### 3.2.2. Experimental Studies

Several experimental studies showed that there are protective effects of lutein against age-related cataracts. For example, a study found that higher quantities of lutein were identified in the cortex’s epithelial layer when compared to the center sections of the lens, which indicated a correlation between lutein concentration and the risk of cataracts in different areas [115]. Moreover, a study found that lutein reduced the levels of protein carbonyl and MDA and lowered DNA damage in H_2_O_2_-induced human lens epithelial cells, suggesting that lutein could protect against cataracts by its antioxidant potential [60]. Additionally, lutein and water chestnut extract reduced the lens opacity in rats with cataracts and increased peroxiredoxin 6 and catalase expression both in rats with cataracts and human lens epithelial cells [61]. Additionally, lutein could also protect the eyes from blue light by its filtering function. For example, a study also found that lutein showed protective effects by suppressing the activation of c-Jun N-terminal kinase (JNK) and p38 in ultraviolet B-induced human lens epithelial cells [62]. An experimental study performed short-term and long-term lutein administration to type 1 diabetic rats and found that lutein inhibited N-epsilon-(carboxymethyl)lysine and N-epsilon-(carboxyethyl)lysine and inhibited the progression of cataractogenesis in the lens of diabetic rats [63]. The details of experimental studies are shown in Table 2.

In summary, several studies suggested that lutein protected against cataracts through its function of blue light filtering and antioxidant potential.

#### 3.2.3. Clinical Trials

A randomized, controlled, double-blind trial in seventeen patients with age-related cataracts found that lutein supplementation for 2 years improved visual performance, while α-tocopherol supplementation did not show the same effect [106]. Another clinical study found that lutein improved visual acuity and glare sensitivity with lutein supplementation to subjects with cataracts and AMD for 13 months on average [107]. Moreover, a study found that lutein supplement for 6 weeks after cataract surgery increased ROS scavenging activities in cataract patients [108]. However, the Age-Related Eye Disease Study 2 revealed that lutein/zeaxanthin supplementation in individuals showed no protective effects on progression to cataracts, but there was a protective effect in individuals who were in the lowest quintile of lutein/zeaxanthin dietary intake (HR = 0.68, 95% CI = 0.48–0.96, *p* = 0.03) [109].

In summary, lutein supplementation could improve visual performance in patients with cataracts and prevent cataracts in individuals, especially in the population who did not have enough lutein in their diet.

### 3.3. Lutein and Alzheimer’s Disease

**Figure 3:**
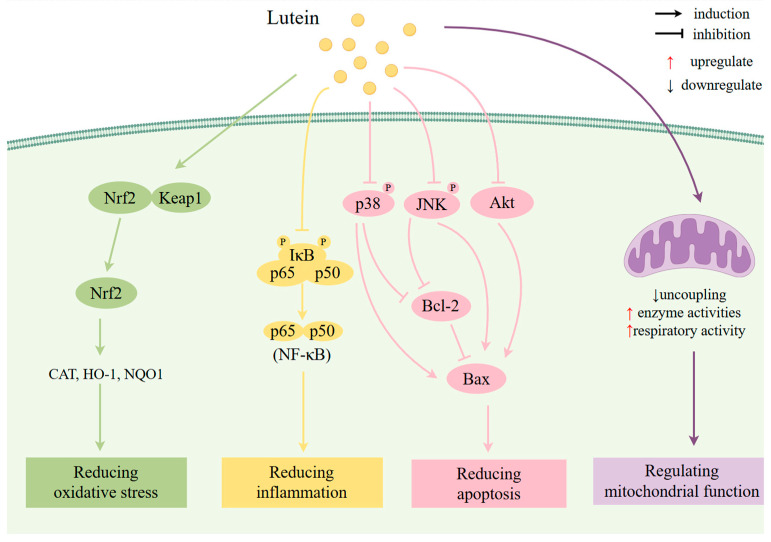
Effects and mechanism of lutein on Alzheimer’s disease. Underlying mechanisms are activation of Nrf2 pathway; downregulation of NF-κB pathway; downregulation of ERS, p38 pathway, and Akt pathway; and regulation of mitochondrial function. Akt, serine/threonine protein kinase; Bax, BCL2-associated X; Bcl-2, B-cell lymphoma-2; CAT, catalase; HO-1, heme oxygenase 1; IκB, inhibitor of NF-κB; JNK, c-Jun N-terminal kinase; Keap1, Kelch-like ECH-associated protein 1; NQO1, NAD(P)H quinone oxidoreductase 1; Nrf2, nuclear factor erythroid 2-related factor 2; NF-κB, nuclear factor kappa-B. Figure by Figdraw.com (accessed on 27 July 2024).

Alzheimer’s disease (AD) is a neurological condition that worsens with time and is usually associated with cognitive decline and memory loss [116]. In the research of AD treatments, stem cell therapies have shown the potential to restore impaired neurons and slow AD progression [117]. Lutein has shown its potential to improve cognitive function, prevent AD, and delay AD progression in multiple studies [118]. The mechanisms of lutein in AD are discussed in detail below (Figure 3 and Table 2).

#### 3.3.1. Epidemiological Studies

Several epidemiological studies revealed that a higher lutein intake was correlated with a reduced risk of AD. For example, a cross-sectional study on donated brains found that the brains of AD patients had lower levels of lutein and higher levels of xanthophyll metabolite compared to healthy elderly brains [27]. Another case–control study revealed that AD patients had significantly lower lutein concentrations of red blood cells than control subjects and higher peroxidized phospholipid concentrations [28]. Moreover, the fasting plasma carotenoid level was much lower in moderately severe AD patients than in mild AD patients and control subjects in a case–control study [29]. A cohort study found that higher lutein concentration in plasma was significantly related to a lower risk of all-cause AD (HR = 0.759, 95% CI = 0.600–0.960, *p* = 0.021) based on 1092 older participants without dementia for 10 years [26]. An analysis of the Third Nutrition and Health Examination Survey (NHANES III) database and the NHANES III Linked Mortality File showed that higher serum levels of lutein/zeaxanthin at baseline were correlated with reduced AD mortality (HR = 0.43, 95% CI = 0.22–0.85) [30]. Furthermore, a meta-analysis of 52 case–control studies revealed that lutein and other antioxidant concentrations in the plasma of AD patients were significantly lower [31]. Another meta-analysis of sixteen studies with 10,633 participants discovered that the levels of lutein in plasma or serum were much lower in patients with AD versus cognitively intact controls [32]. The results of some epidemiological studies are shown in Table 1.

In brief, several epidemiological studies have discovered that higher lutein concentrations in brain tissue, plasma, and red blood cells are associated with lower risks of AD.

#### 3.3.2. Experimental Studies

Numerous studies have demonstrated the protective effects of lutein against AD, with antioxidation and anti-inflammation serving as the primary mechanisms of action [119], which will be discussed below. The details of experimental studies are shown in Table 2.

##### Antioxidant Effects

Oxidative stress is an important factor in AD development [120]. Lutein, as an antioxidant, has shown antioxidative functions against AD in many experimental studies. For example, a study found that lutein improved passive avoidance learning and spatial and cognitive memory, decreased MDA and total oxidant status levels, and increased total antioxidant capacity levels in amyloid beta (Aβ)-induced rats [64]. Another study showed that oral lutein supplementation ameliorated AD by reducing lipid peroxidation in scopolamine-induced mice and zebrafish [65]. Furthermore, one study revealed that rats with the combination of exercise and lutein/zeaxanthin showed reduced levels of lipid peroxidation, increased levels of antioxidant enzyme activities, and the upregulation of Nrf2 and HO-1 [66]. Also, a study discovered that lutein pretreatment showed improvement in cell viability and reduced levels of ROS and lipid peroxidation with upregulated Nrf2 expressions in Aβ peptide-treated bEND.3 cells, indicating that lutein may protect against AD through its antioxidant effects [67].

##### Anti-Inflammatory Effects

Neuroinflammation is the hallmark of AD and plays a vital role in its pathophysiology [121]. Several experimental studies indicated that lutein could reduce inflammation in brain tissues. For example, a study showed that lutein increased the secretion of anti-inflammatory cytokine interleukin (IL)-10 and decreased the secretion of pro-inflammatory cytokine tumor necrosis factor α (TNF-α) in H_2_O_2_-treated BV-2 cells [68]. A study in glutamate-treated SH-SY5Y cells also found that lutein reduced the levels of ROS and CAT enzyme activity; increased SOD enzyme activity; downregulated TNF-α, IL-6, and IL-8 cytokine secretions; and inhibited iron accumulation and lipid peroxidation [69]. Moreover, many studies found that lutein downregulated the NF-κB pathway, which may be the mechanism of its anti-inflammatory potential [64,67].

##### Anti-Apoptosis Effects

Apoptosis contributes to the pathogenesis of AD [122], and many studies revealed the anti-apoptosis function of lutein in many experiments. For example, a study found that pretreatment with lutein extract from silk in cultured rat PC12 cells inhibited the generation of ROS, apoptosis, the activation of the mitogen-activated protein kinase (MAPK) pathway, and reduced the loss of cell viability caused by Aβ [70]. Moreover, a study discovered that lutein treatment reduced the death of nigral dopaminergic neurons; prevented the activation of Bax and caspase-3, -8, and -9; and increased the expression of Bcl-2 to suppress neuronal damage and apoptosis and reduce oxidative stress in 1-methyl-4-phenyl-1,2,3,6-tetrahydropyridine (MPTP)-induced male C57BL/6 mice [71]. Another study found that lutein treatment decreased oxidative stress and ROS generation in PC12 cells exposed to methylglyoxal. It also inhibited mitochondrial damage and cell apoptosis with higher Bcl-2/Bax and a lower mRNA expression of *Bax*, *caspase-3* and *caspase-9*, as well as higher mRNA expression of *Bcl-2*, *PI3K*, and *Akt*, indicating that the phosphoinositide 3-kinase/protein kinase B (PI3K/Akt) signaling pathway could be a possible mechanism [72]. Another study in H_2_O_2_-induced PC12 cells found that the combination of lutein and docosahexaenoic acid (DHA) treatment inhibited the mRNA expression of AD-related gene *AMAD10* and gene *Bax* and inhibited the phosphorylation of JNK1/2, which contributed to the MAPK pathways [73].

###### Regulation of Mitochondrial Function

Mitochondrial dysfunction is closely related to the pathogenesis of AD. It leads to energy exhaustion, oxidative stress, calcium overload, and the activation of caspases, which are the main causes of neuronal dysfunction [123]. It was found that lutein reduced neuronal damage and ameliorated mitochondrial dysfunction and motor abnormalities in MPTP-induced male *C57BL/6* mice [71]. Another study showed that the delivery of lutein and zeaxanthin over 24 h in vitro protected against mitochondrial uncoupling but did not restore ATP production in oxidized phospholipid 1-palmitoyl-2-(5′-oxo-valeroyl)-sn-glycero-3-phosphocholine (POVPC)-induced SH-SY5Y cells [74]. In addition, *nuo-5/*NDUFS1- and *lpd-5/*NDUFS4-depleted *C. elegans* were shown to overexpress synaptic neuroligin as a consequence of mitochondrial dysfunction, and lutein restored neuroligin expression [75]. Moreover, after 14 days of lutein administration in rats treated with 3-nitropropionic acid, there was a significant improvement in body weight, neurobehavioral changes, and oxidative stress and an enhancement in mitochondrial enzyme complex activities [76]. Furthermore, treatment with lutein was found to promote the differentiation of SH-SY5Y cells, particularly enhancing the expression of microtubule-associated protein 2 and neuronal arborization, and the neuronal differentiation was possibly mediated via the regulation of PI3K pathway-induced mitochondrial respiration and signaling [77].

##### Other Mechanisms

The accumulation of toxic Aβ plaques, which is caused by the misregulated proteolytic activity of amyloid precursor protein (APP) and thought to be the hallmark of AD, results in intracellular accumulation and the start of a series of events that eventually lead to neuron damage [124]. A study found that the carotenoid fraction from apricot powerfully showed the inhibition of Aβ fibril formation and fibril-destabilizing effects, and lutein showed the strongest inhibitory effect on Aβ fibril formation [78]. Chemistry and molecular docking analysis also revealed possible van der Waals interactions and hydrogen bonding that may exist between lutein and Aβ, suggesting that lutein could inhibit Aβ aggregation [125]. Additionally, a study found that the oral administration of lutein reversed memory deficit mainly through inhibiting the increase in acetylcholinesterase activity in ethanol-induced Wistar rats [79].

In summary, several studies suggested that lutein protects against AD through reducing the oxidative stress level, increasing anti-inflammatory capacity, inhibiting apoptosis in nervous cells, improving mitochondrial dysfunction, and inhibiting the aggregation of Aβ and the activities of acetylcholinesterase.

#### 3.3.3. Clinical Trials

A randomized, double-blind, placebo-controlled trial found that individuals with self-reported cognitive problems supplemented with 10 mg lutein and 2 mg zeaxanthin for six months showed improved visual memory and learning ability [111]. One randomized controlled trial revealed that taking lutein and zeaxanthin supplements for a year ameliorated cognitive decline in the verbal learning task in older persons [126]. In a randomized, double-blind clinical trial, individuals with AD and controls were given carotenoids (10 mg meso-zeaxanthin, 10 mg lutein, 2 mg zeaxanthin per day) or placebo for six months. The results showed that carotenoid significantly improved the serum concentrations of lutein, zeaxanthin, meso-zeaxanthin, and MP, and there was a significant difference in the dietary intake of lutein/zeaxanthin between the two groups. However, none of the evaluated outcome factors related to cognitive function showed any significant changes, which may be caused by the short period of intervention and the stage of patients [110]. A clinical trial in AD patients and healthy control subjects found that AD subjects had higher serum 1-palmitoyl-2(5′-oxo-valeroyl)-sn-glycero-3-phosphocholine (POVPC) and lower ferric-reducing antioxidant potential compared to controls, and after receiving the same supplement as above for six months, AD patients’ serum POVPC did not differ from that of healthy controls [112]. The results of some clinical trials are shown in Table 3.

In summary, lutein supplementation could improve cognitive function, visual memory, and learning ability in older people.

### 3.4. Lutein and Parkinson’s Disease

Parkinson’s disease (PD) is a progressive age-related neurodegenerative disease with multiple motor symptoms (rigidity, tremors, and bradykinesia) and non-motor symptoms (mood changes and sleep disorders). In the progression of PD, the imbalance of ROS causes the accumulation of Lewy bodies, which eventually leads to mitochondrial dysfunction and cell apoptosis [127]. The effects of lutein on PD have been studied, which will be discussed in detail below.

#### 3.4.1. Epidemiological Studies

Epidemiological studies on the effect of lutein on PD are contradictory. A cohort study found that a higher lutein/zeaxanthin intake was associated with a slower progression of PD signs in 682 individuals without PD at baseline [33]. However, another cohort study from the Singapore Chinese Health Study found that there was no association between lutein consumption and the risk of PD [34]. A case–control study in 1999 found that a higher lutein intake was associated with a higher PD risk (OR = 2.52, 95% CI = 1.32–4.84) [35]. Moreover, a meta-analysis in cohort studies and case–control studies discovered that lutein intake was positively correlated with the risk of PD, and no dose–response association was observed between lutein intake and the risk of PD [36]. The details of epidemiological studies are shown in Table 1.

In brief, the epidemiological evidence of the use of lutein in Parkinson’s disease is inconsistent. This could be because of the differences in race, sample size, and study design. In the future, more epidemiological studies with a bigger sample size, stricter study design, and representative groups should be conducted.

#### 3.4.2. Experimental Studies

A study discovered that lutein treatment reduced the loss of nigral dopaminergic neurons; prevented the activation of Bax and caspase-3, -8, and -9; and increased the expression of Bcl-2 to suppress neuronal damage and apoptosis and reduce oxidative stress in MPTP-induced male C57BL/6 mice, suggesting that lutein could protect against PD through exhibiting antioxidant potential, regulating mitochondrial function, and inhibiting cell apoptosis [71]. Another study used rotenone-induced *Drosophila melanogaster* as a PD model and found that lutein administration prevented the decrease in survival rate and locomotor impairment and restored oxidative stress markers, TH and acetylcholinesterase activity, and dopamine levels [80].

In summary, experimental studies suggested that lutein has protective effects on PD, by reducing the ROS level and inhibiting cell apoptosis. The details of experimental studies are shown in Table 2.

### 3.5. Lutein and Osteoporosis

**Figure 4:**
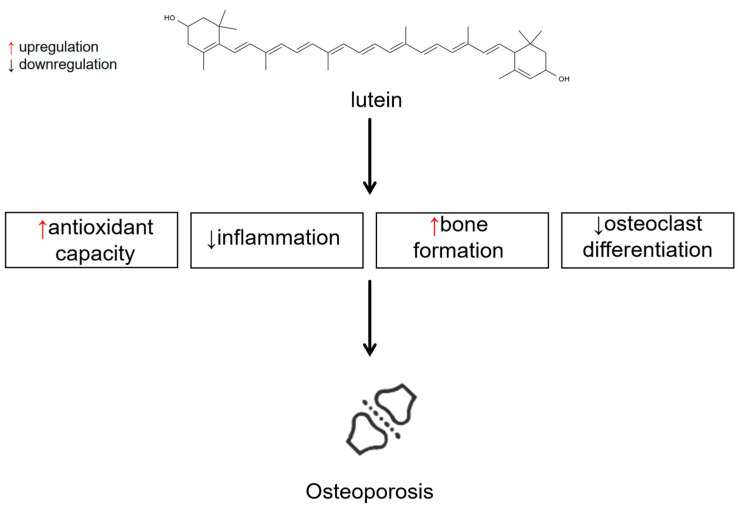
Effects and mechanisms of lutein on osteoporosis. Mechanisms involved are upregulation of antioxidant capacity, downregulation of inflammation, and regulation of bone formation and osteoclast differentiation (accessed on 27 July 2024).

Osteoporosis is a bone disease defined by a decrease in bone mass and abnormalities in the microarchitecture of bone tissues, which will lead to a reduction in bone strength and an elevated risk of fractures. Lutein has exhibited anti-osteoporotic potential and bone-building effects in multiple studies [128]. The effects and mechanisms of lutein on osteoporosis are given in Figure 4 and Table 2.

#### 3.5.1. Epidemiological Studies

Many epidemiological studies have revealed that lutein consumption is closely correlated with bone health and osteoporosis. For example, a cohort study discovered that a higher dietary lutein/zeaxanthin intake was associated with a better bone density status and a lower risk of wrist fracture in women [37]. Also, the Singapore Chinese Health Study with a follow-up cohort of 9.9 years revealed that the consumption of lutein and zeaxanthin was negatively correlated with the risk of hip fractures in men with a lower body mass index [38]. A cohort study of National Health and Nutrition Examination Survey (NHANES) found that there was a marginally significant correlation between a high lutein/zeaxanthin intake and a reduced risk of osteoporosis [39]. Additionally, the oxidative balance status was found to be associated with BMD, and a cross-sectional study assessed the oxidative balance score (OBS) with pro- and antioxidant components classified as non-dietary pro-oxidants, non-dietary antioxidants, dietary pro-oxidants, and dietary antioxidants, including lutein, and discovered that subjects who had a higher OBS had a reduced risk of lumbar spine osteoporosis [40]. A cross-sectional study assessed the relationship of serum lutein/zeaxanthin, MPOD, and bone density in young healthy adults and discovered that the proximal femur and lumbar spine’s bone density were positively correlated with MPOD [41]. Similarly, Framingham Osteoporosis Study discovered that there were no cross-sectional correlations between carotenoid intake and BMD, but longitudinal analyses showed that lutein/zeaxanthin intake was inversely related to the 4-year change in trochanter BMD in elderly men [42]. Additionally, a cross-sectional study revealed that there was no significant association between the serum lutein/zeaxanthin level and bone mineral density (BMD) in a Chinese population, and this was probably due to the difference in lutein/zeaxanthin distribution in tissues [43]. The results of epidemiological studies are given in Table 1.

In brief, most epidemiological studies suggested that a higher lutein intake was associated with higher BMD and a lower risk of osteoporosis and fracture.

#### 3.5.2. Experimental Studies

Experimental studies showed that lutein could ameliorate osteoporosis through antioxidant and anti-inflammatory effects, stimulating bone formation, and suppressing osteoclastic bone resorption, which will be discussed below. The details of experimental studies are shown in Table 2.

##### Antioxidant Effects and Anti-Inflammatory Effects

A study found that lutein treatment reduced lipid peroxidation, downregulated the ROS level, upregulated the Nrf2-induced expressions of antioxidant genes, and downregulated inflammation and osteoclast-specific marker (NFATc1) expression in ovariectomized mice [81]. Another study showed that lutein significantly protected primary chondrocyte cells against monosodium iodoacetate-induced oxidative stress, inflammation, and apoptosis by activating the NF-κB and Nrf2 pathways [82].

##### Other Mechanisms

A study found that lutein promoted mineralized bone nodules in osteoblast cultures and inhibited l, 25-dihydroxyvitamin D3-induced bone resorption and osteoclast formation. Lutein also downregulated the soluble receptor activator of NF-κB (RANK) ligand, which could induce osteoclast formation in bone marrow macrophages [83]. This study also showed that 4-week lutein administration increased the cortical bone mass in the femur of male mice by inhibiting bone resorption and promoting bone formation [83]. Lutein treatment in osteoclasts showed the suppression of osteoclast differentiation and bone resorption induced by IL-1, reduction in mature osteoclasts, inhibition of sclerostin expression, and enhancement in the formation of mineralized bone nodules by elevating bone morphogenetic protein 2 (*BMP2*) mRNA expression [84]. Another study showed that lutein reduced the differentiation and tartrate-resistant acidic phosphatase activity of osteoclasts. However, the expression of RANK mRNA significantly increased when lutein was administered at higher concentrations [85]. Furthermore, a high concentration of lutein treatment exhibited the effect of downregulating metaphyseal alkaline phosphatase activity, but there was no significant influence on bone calcium content in the tissues of young rats in vitro [86].

In summary, current experimental studies supported that lutein had protective potential against osteoporosis through reducing the ROS level, suppressing osteoclast differentiation, increasing bone formation, and decreasing bone resorption.

## 4. Bioavailability Improvement in Lutein

Because lutein could not be independently synthesized by humans, it is best to obtain lutein from food resources. Foods rich in lutein are green-leaf vegetables and egg yolk. The bioavailability of lutein in egg yolk is high because of its high fat content [129]. Moreover, according to the lipophilic nature of lutein, there are multiple approaches to improve the bioavailability of lutein in green leafy vegetables. For example, its bioavailability can be increased by combining foods high in lutein (such as green leafy vegetables) with dietary fats including flaxseed oil, olive oil, and cow ghee [130,131,132]. Lutein-fortified milk is also a low-cost approach to improving its bioavailability [133]. Additionally, some delivery systems have been proven to increase bioavailability, such as nanoparticle capsules and self-emulsifying phospholipid suspension [134,135,136]. More studies should be conducted to improve the food matrix’s lutein absorption.

## 5. Conclusions and Perspectives

Lutein shows protective potential against aging and age-related diseases. Epidemiological studies discovered that a higher lutein intake was associated with a lower mortality and risk of AMD, cataracts, AD, PD, and osteoporosis. Experimental studies found that lutein exhibited protective function against aging through improving the oxidative stress status and downregulating inflammation. Moreover, lutein showed anti-AMD effects by its antioxidant effects, protective action against light irradiation, and reducing the level of harmful substances and ER stress. Lutein also showed anti-cataract effects by its antioxidant effects and light filtering. Additionally, lutein plays a vital role in preventing AD and improving cognitive functions through antioxidant, anti-inflammatory, and anti-apoptosis effects; the regulation of mitochondrial function; reducing the level of harmful substances in brain tissue; and downregulating acetylcholinesterase activity. Lutein could protect against PD through its antioxidant effects. Furthermore, lutein could prevent osteoporosis by its antioxidant effects, regulation of bone formation and bone resorption, and reducing the differentiation of osteoclasts. Clinical trials also revealed that lutein supplementation is important for the prevention and management of these issues, such as preventing AMD, slowing the progression of AMD, improving visual performance in AMD and patients with cataracts, and improving the cognitive function of AD patients. Additionally, the best way for humans to obtain lutein is from food resources, and the bioavailability of lutein can be greatly increased by consuming high-fat content food at the same time. In the future, more epidemiological studies with a stricter research design, bigger sample size, and representative groups should be conducted because inconsistent results have been reported. In experimental studies, the underlying mechanisms of lutein on aging and age-related diseases should be explored further. Moreover, more clinical studies have to be carried out to validate the effects of lutein on aging and age-related diseases from preclinical studies, and more clinical trials should focus on lutein’s effect on osteoporosis. In addition, this paper is helpful for the public to select foods rich in lutein and for lutein to be developed into functional foods and drugs for the prevention and treatment of aging and age-related diseases.

## Figures and Tables

**Table 1:** Epidemiological studies of lutein on aging and age-related diseases.

Study Type	Participants	Effects	Ref
Aging			
Cross-sectional cohort study	2007 Australian individuals aged 45 to 86	Independently associated with leukocyte telomere length β = 0.079, *p* = 0.03, adjusted for age and sex β = 0.107, *p* = 0.01, additional adjustment for BMI and VO_2_max β = 0.12, *p* = 0.006, further adjustment for vascular risk factors	[12]
Cross-sectional study	5056 American elderly people over the age of 60 years from NHANES	Significantly correlated with increased S-klotho concentration (β = 15.44, *p* < 0.01)	[13]
Umbrella review	29 outcomes in 24 systematic reviews and meta-analyses	Had beneficial effects on age-related cataracts, age-related macular degeneration	[14]
AMD			
Matched case–control study	164 cases of AMD and 164 controls	Associated with decreased risk of AMD (OR = 0.21, 95% CI = 0.05–0.84)	[15]
Meta-analysis	9 studies	Participants in highest category had 47% lower risk of developing AMD (OR = 0.53, 95% CI = 0.40–0.72, *p* < 0.001; I^2^ = 43.3%, *p* heterogeneity = 0.079)	[15]
Cross-sectional study	34 patients with unilateral wet AMD and 33 patients with bilateral dry AMD	Patients with unilateral wet AMD had significantly higher levels of MPOD in their fellow eye but had lower levels compared with patients with bilateral dry AMD (0.58 versus 0.48, *p* = 0.026)	[16]
Cohort study	609 participants	Participants with higher plasma lutein had reduced risk for incident advanced AMD in fully adjusted model (HR = 0.63 per 1 SD increase (95% CI = 0.41–0.97), *p* = 0.03)	[17]
Case–control study	260 AMD cases and 260 matched controls	Lutein was associated with lower AMD risk (OR = 0.30, 95% CI = 0.10–0.88) comparing extreme quartiles	[18]
Cohort study	63,443 women and 38,603 men (Nurse study)	Pooled relative risk comparing extreme quintiles (HR = 0.59; 95% CI = 0.48–0.73; *p* for trend < 0.001)	[19]
Case–control study	158 participants with AMD and 50 participants without AMD	No significant difference between AMD and non-AMD group	[20]
Cataracts			
Cross-sectional study	1689 subjects aged 61–80 years	Lutein was associated with lower nuclear cataract risk (RR = 0.58, 95% CI = 0.35–0.98, *p* = 0.041)	[21]
Cohort study	478 women without diabetes aged 53 to 73	Lutein intake was inversely associated with risk of nuclear opacification, comparing each quintile	[22]
Meta-analysis	1 cohort study and 7 cross-sectional studies	Lutein concentration in blood was inversely associated with risk of nuclear cataracts (pooled RRs = 0.63, 95% CI = 0.49–0.77)	[23]
Meta-analysis	6 cohort studies	Lutein and zeaxanthin intake was inversely associated with risk of nuclear cataracts (RR = 0.75, 95% CI = 0.65–0.85); every 300 µg/d increase in dietary lutein and zeaxanthin intake was linked to 3% decrease in risk of nuclear cataracts	[24]
Meta-analysis	8 RCTs and 12 cohort studies	Dietary lutein/zeaxanthin was inversely correlated with risk of age-related cataracts (RR = 0.81, 95% CI = 0.75–0.89, *p* < 0.001), and dose–response analysis found that every 10 mg/d increase in dietary lutein and zeaxanthin intake was linked to 26% decrease in risk of age-related cataracts	[25]
AD			
Cohort study	1092 older participants without dementia	Lutein was associated with decreased risk of all-cause AD (HR = 0.759, 95% CI = 0.600–0.960, *p* = 0.021, for +1 SD)	[26]
Cross-sectional study	21 AD brains and 10 healthy brains	AD brains had significantly lower levels of lutein (*p* = 0.04)	[27]
Case–control study	28 control subjects (age: 74.1 ± 1.3 years) and 28 patients with AD (age: 72.5 ± 1.4 years)	Concentrations of RBC lutein in AD patients were significantly lower than in control subjects. (*p* < 0.001) Inverse relationship was seen between RBC lutein and antioxidant concentrations (*p* < 0.05) in AD patients	[28]
Case–control study	36 AD subjects and 10 control subjects	Lutein was significantly correlated with MMSE	[29]
Cohort study	6958 participants aged older than 50 years	Lutein was associated with lower risk of AD mortality (HR = 0.43, 95% CI = 0.22–0.85), highest quartile compared to lowest quartile	[30]
Meta-analysis	52 case–control studies	AD patients had significantly lower plasma levels of lutein (*p* = 0.01, I^2^ = 88%)	[31]
Meta-analysis	16 studies, with 10,633 participants	AD Patients had significantly lower plasma/serum levels of lutein (SMD = −0.86, 95% CI = −1.67 to −0.05, *p* = 0.04)	[32]
PD			
Cohort study	682 participants without Parkinson’s disease	Lutein/zeaxanthin intake was inversely associated with rate of progressive Parkinsonian signs (β = −0.05, 95% CI = −0.09 to −0.02)	[33]
Cohort study	63,257 men and women aged 45 to 74 years	No association between lutein consumption and risk of Parkinson’s disease	[34]
Case–control study	126 Parkinson’s disease cases and 432 controls	Higher lutein intake was associated with higher Parkinson’s disease risk, comparing extreme quartiles (OR = 2.52, 95% CI = 1.32–4.84)	[35]
Meta-analysis	6 cohort studies, 2 nested case–control studies, and 6 case–control studies	Lutein intake was positively associated with risk of Parkinson’s disease (RR = 1.86, 95% CI = 1.20, 2.88) in case–control studies; no dose–response correlation was found between lutein intake and risk of Parkinson’s disease	[36]
Osteoporosis			
Cohort study	EPIC-Norfolk, *n* = 25,439	Lutein had positive trends in BUA bone density for women across quintiles (*p* = 0.01); lutein was associated with lower risk for wrist fracture in women across quintiles (*p* = 0.022)	[37]
Cohort study	63,257 men and women (age: 45–74 years)	Dietary lutein/zeaxanthin was negatively correlated with men’s risk of hip fractures (*p* = 0.049)	[38]
Cohort study	4820 NHANCES participants	Dietary lutein/zeaxanthin intake was associated with reduced risk of osteoporosis (OR for quintile 5 vs. 1 = 0.53; 95% CI = 0.30–0.94; *p* for trend = 0.076)	[39]
Cross-sectional study	151 postmenopausal Iranian women aged 50–85 years old	Highest tertile of OBS had lower risk of lumbar spine osteoporosis than those in lowest tertile (OR = 0.14; 95% CI = 0.04–0.45; *p* = 0.001)	[40]
Cross-sectional study	63 subjects (females, *n* = 39; males, *n* = 24; average age = 22.5 years old)	MPOD was positively correlated with proximal femur and lumbar spine’s bone density (*p* < 0.05)	[41]
Cohort study	5209 men and women aged 28–62 years old	No cross-sectional correlations between dietary lutein/zeaxanthin intake and BMD. Dietary lutein/zeaxanthin intake was inversely related to 4-year change in trochanter BMD in elderly men (*p* for trend = 0.008)	[42]
Cross-sectional study	1898 women and 933 men aged 59.6 years	No significant association between serum lutein/zeaxanthin level and BMD	[43]

Abbreviations: AD, Alzheimer’s disease; AMD, age-related macular degeneration; BMD, bone mineral density; BUA, broadband ultrasound attenuation; HR, hazard ratio; MMSE, minimum mental state examination score; MPOD, macular pigment optical density; OBS, oxidative balance score; OR, odds ratio; RBC, red blood cell; SD, standard deviation; S-klotho, soluble klotho; 95% CI, 95% confidence interval.

**Table 3:** Clinical trials of lutein on aging and age-related diseases.

Study Type	Subjects	Substance and Dose	Duration	Effects	Ref.
AMD					
Randomized, double-blinded, placebo-controlled trial	112 early AMD patients	10 mg or 20 mg lutein, or a combination of lutein (10 mg) and zeaxanthin (10 mg)	2 years	↑ serum lutein concentration and MPOD ↑ contrast sensitivity	[100]
Randomized, double-blinded, placebo-controlled trial	Participants with probable AMD who were 50 to 79 years of age (*n* = 108)	10 mg or 20 mg lutein, or a combination of lutein (10 mg) and zeaxanthin (10 mg)	2 years	↑ MPOD ↑ contrast sensitivity	[101]
Randomized, double-blind, placebo-controlled, two-center trial	72 patients (mean age 70.5 ± 8.7)	10 mg lutein	4 months	↑ MPOD ↑ visual acuity in the subgroup that had worse visual acuity	[102]
Multicenter, randomized, double-blinded, placebo-controlled phase 3 study	4203 participants aged 50 to 85 years at risk for progression to advanced AMD	Lutein (10 mg) + zeaxanthin (2 mg), or DHA (350 mg) + EPA (650 mg), or combination of lutein + zeaxanthin and DHA + EPA, or placebo.	Median follow-up = 5 years	No significant reduction in progression to advanced AMD	[103]
Randomized (2:1), placebo-controlled, double-masked parallel group study	126 patients with AMD	In months 1 to 3, dose was 20 mg lutein once daily, and in months 4 to 6, dose was 10 mg lutein once daily	6 months	↑ MPOD No significant effect of lutein supplementation on VA or macular function; significant correlation was found between increase in MPOD after 6 months and increase in MDLT and VA after 6 months	[105]
Cataract					
Randomized, double-blind, controlled clinical trial	17 patients clinically diagnosed with age-related cataracts	15 mg lutein, three times a week	2 years	↑ serum concentrations of lutein ↑ visual acuity and glare sensitivity	[106]
Clinical trial	10 subjects diagnosed with cataracts or age-related macular degeneration	12 mg of all-trans-lutein, 3 mg of 13/15-cis-lutein, and 3.3 mg of α-tocopherol	26 months on average	↑ serum concentration of lutein ↑ visual acuity and glare sensitivity	[107]
Clinical trial	40 patients with cataracts	Multiple antioxidants, including 6 mg lutein	6 weeks	↑ superoxide scavenging activity ↑ H_2_O_2_ ↓ hydroperoxides	[108]
Multicenter, double-blind clinical trial	4203 participants, aged 50 to 85 years	Lutein/zeaxanthin for 10 mg/2 mg	4.7 years on average	↓ risk of progression to cataract surgery	[109]
AD					
Randomized, double-blind, controlled clinical trial	31 AD patients and 31 control subjects	10 mg meso-zeaxanthin, 10 mg lutein, and 2 mg zeaxanthin per day	6 months	No significant changes in any of cognitive function outcome variables measured	[110]
Randomized, double-blind, placebo-controlled trial	90 volunteers aged 40–75 years	10 mg of lutein and 2 mg of zeaxanthin	6 months	↑ visual episodic memory ↑ visual learning	[111]
Randomized, double-blind, placebo-controlled trial	AD patients (*n* = 21) and healthy age-matched control subjects (*n* = 16)	10 mg meso-zeaxanthin, 10 mg lutein, and 2 mg zeaxanthin	6 months	Novel oxidized phospholipid biomarker POVPC levels of AD patients were not different compared to healthy controls No significant effect on cognitive performance	[112]

Abbreviations: AD, Alzheimer’s disease; AMD, age-related macular degeneration; MDLT, mean differential light threshold; MPOD, macular pigment optical density; POVPC, 1-palmitoyl-2-(5-oxovaleroyl)-sn-glycero-3-phosphocholine; VA, visual acuity. ↑, improvement; ↓, reduction.
